# Microbiome-Mediated Immune Signaling in Inflammatory Bowel Disease and Colorectal Cancer: Support From Meta-omics Data

**DOI:** 10.3389/fcell.2021.716604

**Published:** 2021-11-16

**Authors:** Molly Pratt, Jessica D. Forbes, Natalie C. Knox, Charles N. Bernstein, Gary Van Domselaar

**Affiliations:** ^1^ Department of Medical Microbiology and Infectious Diseases, University of Manitoba, Winnipeg, MB, Canada; ^2^ Department of Laboratory Medicine and Pathobiology, University of Toronto, Toronto, ON, Canada; ^3^ National Microbiology Laboratory, Public Health Agency of Canada, Winnipeg, MB, Canada; ^4^ Department of Internal Medicine, University of Manitoba, Winnipeg, MB, Canada; ^5^ IBD Clinical and Research Centre, University of Manitoba, Winnipeg, MB, Canada

**Keywords:** gut microbiome, inflammatory bowel diesases, colorectal cancer, metagenomics, metaproteomics, metabolomics

## Abstract

Chronic intestinal inflammation and microbial dysbiosis are hallmarks of colorectal cancer (CRC) and inflammatory bowel diseases (IBD), such as Crohn’s disease and ulcerative colitis. However, the mechanistic relationship between gut dysbiosis and disease has not yet been fully characterized. Although the “trigger” of intestinal inflammation remains unknown, a wealth of evidence supports the role of the gut microbiome as a mutualistic pseudo-organ that significantly influences intestinal homeostasis and is capable of regulating host immunity. In recent years, culture-independent methods for assessing microbial communities as a whole (termed meta-omics) have grown beyond taxonomic identification and genome characterization (metagenomics) into new fields of research that collectively expand our knowledge of microbiomes. Metatranscriptomics, metaproteomics, and metabolomics are meta-omics techniques that aim to describe and quantify the functional activity of the gut microbiome. Uncovering microbial metabolic contributions in the context of IBD and CRC using these approaches provides insight into how the metabolic microenvironment of the GI tract shapes microbial community structure and how the microbiome, in turn, influences the surrounding ecosystem. Immunological studies in germ-free and wild-type mice have described several host-microbiome interactions that may play a role in autoinflammation. Chronic colitis is a precursor to CRC, and changes in the gut microbiome may be an important link triggering the neoplastic process in chronic colitis. In this review, we describe several microbiome-mediated mechanisms of host immune signaling, such as short-chain fatty acid (SCFA) and bile acid metabolism, inflammasome activation, and cytokine regulation in the context of IBD and CRC, and discuss the supporting role for these mechanisms by meta-omics data.

## Introduction

The community of microorganisms that inhabit the human gastrointestinal (GI) tract and their collective genetic material are referred to as the gut microbiome. The composition and function of the gut microbiome have been widely implicated in human health and disease, particularly in GI diseases such as inflammatory bowel diseases (IBD) and colorectal cancer (CRC), where specific alteration of the gut microbiome is associated with pathology (Sobhani et al., 2011; Knox et al., 2019b; Kaplan et al., 2019). The global incidence of IBD, including component diseases Crohn’s disease (CD), ulcerative colitis (UC), and IBD-unclassified (IBD-U), has risen considerably in recent decades (Molodecky et al., 2012; Kaplan et al., 2019; El-Matary and Bernstein, 2020). Increasing rates of pediatric-onset IBD in North America are of particular concern not only because disease in younger patients is more extensive and can lead to a range of developmental issues (Carroll et al., 2019) but also because of associated long-term cancer risk (Akimoto et al., 2020; El-Matary and Bernstein, 2020). Chronic intestinal inflammation is a precursor to CRC, and IBD is an established risk factor for developing both early- and late-onset CRC (Akimoto et al., 2020; Gausman et al., 2020).

Recent advances in culture-independent techniques for studying microbial communities have enabled extensive characterization of the healthy human gut microbiome (Human Microbiome Project Consortium, 2012; Méndez-García et al., 2018) and provide context for the host-microbiome interactions within the gut microenvironment. Although perturbations in the microbial community composition—dysbiosis—have been characterized in both IBD and CRC, the cause and effect relationship between inflammation and dysbiosis remains unclear. A variety of other host-mediated factors such as genetic susceptibility, epigenetics, diet, antibiotic use, and smoking status have been associated with IBD or CRC risk or both (Glória et al., 1996; Jostins et al., 2012; Kaplan et al., 2019; Lorzadeh et al., 2021), yet no exact trigger has been identified. The gut microbiome is thought to be directly implicated in the etiopathogenesis of both IBD and CRC (Sellon et al., 1998; Peloquin and Nguyen, 2013); current theories hypothesize that alterations in the normal gut microbiome, caused by some environmental exposure [for example, antibiotic use (Ungaro et al., 2014)], can trigger an inflammatory immune response that persists in the genetically susceptible host (Yang and Jobin, 2017; Knox et al., 2019b; Kaplan et al., 2019; Szamosi et al., 2020).

The intestinal epithelium is a crucial interface for host-microbiome interactions. In a healthy gut, the host’s immune system must be able to recognize and tolerate commensal organisms while retaining its ability to defend against pathogens. For example, microbial taxa that are considered protective stimulate CD4^+^ T regulatory (T_reg_) cell proliferation and maintenance of gut immune homeostasis (Atarashi et al., 2011; Knox et al., 2019b), whereas pathogenic organisms are recognized by Toll-like receptors (TLRs) and NOD-like receptors (NLRs) on CD4^+^ T cells, resulting in a coordinated adaptive immune response (Himmel et al., 2008). Similarly, the gut microbiota responds to host immune activation and local inflammation by altering gene expression and metabolite production (Becattini et al., 2021). Gathering a better understanding of this complex, bi-directional signaling is the basis for untargeted microbiome functional characterization.

**FIGURE 1:**
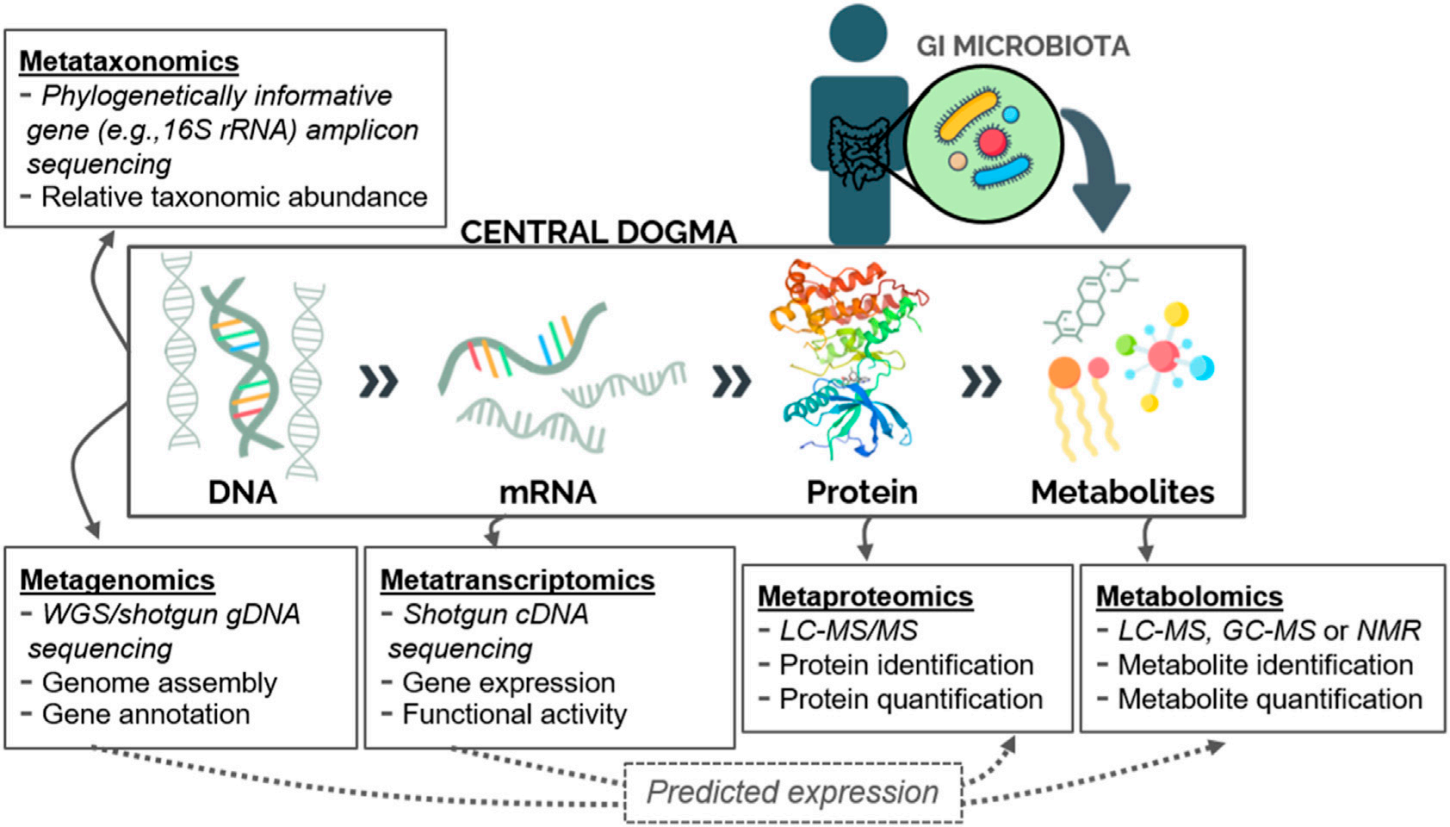
Meta-omics techniques for studying the human gut microbiome. Microbial communities can be characterized based on their collective gene content (metataxonomics or metagenomics), gene transcripts (metatranscriptomics), protein pool (metaproteomics), or metabolite pool (metabolomics). Additionally, sequence data can be used to predict downstream expression of proteins or metabolites (dashed arrows). Abbreviations: WGS: Whole-genome sequencing; LC: Liquid chromatography; MS: Mass Spectrometry; GC: Gas chromatography; NMR: Nuclear magnetic resonance.

Techniques for characterizing entire microbial communities and their physiological contributions (termed meta-omics) have now grown beyond taxonomic identification and genome mapping as studied via metataxonomics and metagenomics, respectively, into new fields of research (Figure 1). Metatranscriptomics, metaproteomics, and metabolomics are omics approaches that allow for further characterization of the gut microbiome in health and disease (Figure 1). Metagenomics describes the genetic content of a microbial community within a sample (typically stool or intestinal biopsy in the case of the GI microbiome), whereas metatranscriptomics utilizes reverse transcription to evaluate gene expression patterns from microbial messenger RNA (mRNA). Both techniques involve shotgun sequencing of nucleic acids isolated from a biological specimen and allow for prediction of downstream functional activity (Figure 1). Metaproteomics generally uses liquid chromatography with tandem mass spectrometry (LC-MS/MS) to isolate and quantify proteins, which are then evaluated to identify potential biomarkers of disease. Lastly, metabolomics can be used to search for a wide range of biomarkers, including amino acids, fatty acids, sugars, and vitamins. Using this approach, metabolites are detected through either nuclear magnetic resonance (NMR) or MS coupled with chromatography, the latter offering a wider range of detection and higher sensitivity. Stool is frequently used as a proxy for the luminal GI microbiome; however, the mucosal-associated microbiome—captured through endoscopic biopsy—will differ from that of the lumen and can provide site-specific evidence. Likewise, many microbiome studies focus on the bacterial component of the microbiome as a proxy for the entire community, which additionally contains eukaryotes and/or viruses. Meta-omics approaches may be used individually or in combination to study the microbiome. The application of multiple techniques to samples from the same cohort yields correlated functional profiles (Lloyd-Price et al., 2019), supporting the usefulness of microbial sequence data for downstream prediction of functional activity (Figure 1). Uncovering particular taxonomic and metabolic contributions in the context of IBD and CRC using these approaches gives insight into the metabolic microenvironment of the gut and the role of microorganisms within that environment. The potential of stool meta-omics techniques as non-invasive screening and detection tools is especially attractive. While this review focuses on microbiome-mediated signaling specifically, meta-omics can also be used to profile and predict disease-specific, location-specific, or longitudinal changes in microbial communities and many such studies have provided valuable insights into microbial community dynamics (Gevers et al., 2014; Hall et al., 2017; Schirmer et al., 2018).

Investigators face several challenges when using meta-omics data for microbiome research as well as for disease investigation in a clinical setting. Prior to data generation there are a number of ways in which bias may be introduced during sample collection and storage (Alberti et al., 2014; Reck et al., 2015; Neuberger-Castillo et al., 2021). Furthermore, due to the dynamic and compositional nature of microbiomes, many of the techniques described above require large sample sizes and result in even larger datasets that must be properly assessed for quality before their interpretation (Zhang et al., 2021a). Accurate reference-based taxonomic assignment of sequences depends on the quality of database that is used and achieving statistical significance, in differential expression analysis for example, can be challenging (Zhang et al*.,*2021b). There is considerable interest in evaluating individual GI microbiomes within a clinical setting to aid screening and diagnosis using a personalized approach (Knox et al., 2019a). However, many of the techniques discussed above require a substantial computational effort and corresponding technical expertise, making them difficult to implement routinely in a clinical setting. Readers are referred to the following discussions regarding specific challenges associated with meta-omics techniques (Wishart 2016; Quince et al., 2017; Shakya et al., 2019; Zhang et al., 2019; Krassowski et al., 2020).

## Meta-Omic Explorations of the Gut Microbiome in IBD and CRC Reveal Common Patterns of Metabolic Dysregulation

The implication of the gut microbiome in CRC and IBD etiology has prompted many researchers to employ meta-omics to study microbiome function and activity (Franzosa et al., 2019; Lloyd-Price et al., 2019; Ocvirk et al., 2020). Indeed, IBD serves as a model disease for the integrative human microbiome project (iHMP), which began in 2014 and collects host and microbiome-associated data using multiple meta-omics strategies (The Integrative HMP (iHMP) Research Network Consortium, 2014; Lloyd-Price et al., 2019). Results from the iHMP and other studies reporting statistically significant changes in the microbiome between health and disease are shown in Table 1. Extensive characterization of CRC-associated (Wirbel et al., 2019) and IBD-associated (Lloyd-Price et al., 2019) microbiomes has demonstrated that although the dysbiotic communities differ on a taxonomic level (*i.e.,* biomarker species), there are similarities between the two diseases at the functional level (*e.g.,* depletion of butyrate-producers, bile acid dysregulation). Consequently, in this review we restrict our discussion to the functional potential and activity of disease-associated microbiomes, rather than focusing on their taxonomic composition.

**TABLE 1:** Meta-omic studies reporting statistically significant changes in the GI microbiome in CRC or IBD. Abbreviations: healthy controls (HC); irritable bowel syndrome (IBS); high-resolution magic angle spinning nuclear magnetic resonance (HR-MAS NMR); gas chromatography (GC); capillary electrophoresis (CE); ion cyclotron resonance Fourier transform mass spectrometry (ICR-FT/MS); time-of-flight mass spectrometry (ToFMS); liquid chromatography (LC); high performance LC (HPLC); ultra-performance LC (UPLC); medium-chain fatty acids (MCFA); long-chain fatty acids (LCFA).

Cohort type (N)	Sample type(s)	Method	Increased in disease	Decreased in disease	References
CRC					
CRC (31): Colorectal tumour biopsy vs. normal tissue	Mucosal biopsy	Metabolomics: HR-MAS NMR and GC/MS	Choline-containing compounds, taurine, scyllo-inositol, lactate, phosphocholine, phosphate, L-glycine, 2-hydroxy-3-methyl valerate, L-proline, L-phenylalanine, palmitic acid, margaric acid, oleic acid, stearic acid, uridine, 11-eicosenoic acid, propyl octadecanoate, cholesterol	Lipids, polyethylene glycol, glucose, fumarate, malate, mannose, galactose, 1-hexadecanol, arachidonic acid	Chan et al. (2009)
CRC (11) vs. HC (10)	Stool, mucosal biopsy	Metabolomics: GC/ToFMS	Uracil, uridine, proline	Fructose, linoleic acid, nicotinic acid, glucose, galactose, 3-phosphoglycerate, citric acid, inosine, creatine	Phua et al. (2014)
Meta-analysis of CRC (386) vs. tumor-free controls (392)	Stool	Metagenomics: WGS	Metabolic modules: amino acid degradation, organic acid metabolism, glycoprotein degradation, bile acid conversion	Metabolic modules: carbohydrate degradation	Wirbel et al. (2019)
CRC stage 0 (73); stage I/II (111); stage III/IV (68); multiple polypoid adenomas (MP, 67); vs. HC (251); normal with history of surgery (HS, 34)	Stool	Metagenomics: WGS, Metabolomics: CE/ToFMS	Taxonomy: sulfide-producing species – *Desulfovibrio vietnamensis, D. longreachensis, Bilophilia wadsworthia* Metabolites: deoxycholate (MP vs. HC); glychocholate, and taurocholate, branched-chain amino acids, phenylalanine, tyrosine and glycine (S0 vs. HC); serine (SIII/IV vs. HC), Pathway gene abundance: amino acid metabolism, sulfide production, phenylalanine and tyrosine biosynthesis (all CRCs vs. HC); sulfate reductase (*dsrA*), cofactor and vitamin biosynthesis, lysine biosynthesis and degradation, methane metabolism (SIII/IV vs. HC)	Taxonomy: butyrate-producing species - *Lachnospira multipara, Eubacterium eligens,* Pathway gene abundance: tryptophan biosynthesis (SIII/IV vs. HC)	Yachida et al. (2019)
Healthy Alaskan Natives (high-risk group for CRC) (32) vs. Healthy Rural Africans (low risk for CRC) (21)	Stool, urine	Metataxonomics: 16S rRNA sequencing, Metabolomics: ^1^H-NMR spectroscopy, GC, HPLC-MS	*Enriched in high-risk population:* Actinobacteria, Verrumicrobia, Lachnospiraceae, *Bifidobacterium spp., Escherichia-Shigella spp.,* choline, formate, cholate, chenodeoxycholate, deoxycholate, conjugated bile acids, nicotinamide/niacin metabolites	*Enriched in low-risk population:* Ruminococcaeceae, Prevotellaceae, *Prevotella 9,* Ruminococcaceae, *Succinivibrio, Eubacterium coprostanoligenes*, amino acids, purines^a^, pyrimidines^a^, butyrate, propionate, nicotinate	Ocvirk et al. (2020)
CRC (14) vs. HC (14)	Stool	Metaproteomics: LC-MS/MS	Desulfobacterales, *Methanobacteriaceae, Sporolactobacillaceae, Bacteroides fragilis, Peptostreptococcus anaerobius,* DNA replication, recombination, and repair proteins, iron intake and transport proteins, superoxide dismutases	*Sutterellaceae, Epulopiscium, Gordonibacter,* NADH:flavin oxidoreductases/NADH oxidases, energy production and conversion proteins, amino acid transport and metabolism, coenzyme transport and metabolism, lipid transport and metabolism, translation machinery, cell wall, membrane, and envelope biogenesis, cell motility, post-translational modification, protein turnover, and chaperones, inorganic ion transport and metabolism	Long et al. (2020)
IBD					
Identical twin pairs (N = 17 pairs): discordant colonic CD*(4p); discordant ileal CD*(2p); concordant ICD*(2p); concordant CCD*(2p) vs. HC (7 pairs), *in remission	Stool	Metabolomics: ICR-FT/MS	Bile acid metabolism^b^ (glycocholate, glycochenodeoxycholate taurocholate, Trihydroxy-6β-cholanate), amino acid metabolism^b^, tyrosine^b^, tryptophan (ICD only), phenylalanine (ICD only), fatty acid biosynthesis (ICD only; oleic acid, stearic acid, palmitic acid, linoleic acid, arachidonic acid), Urea cycle^a^ ^,^ ^b^, vitamin B6 metabolism^b^	Arachidonic acid metabolism/prostaglandins^a^ ^,^ ^b^ (PGs; PGF2a)	Jansson et al. (2009)
CD (83); UC (68); pouchitis (13) vs. HC (40)	Stool	Metabolomics: GC-MS	Styrene^c^	MCFAs, hexanoate^b^, protein fermentation metabolites	De Preter *et al.* (2015)
Paediatric IBD in remission -CD (26); UC(10) vs. healthy 1st-degree relatives (54)	Stool	Metataxonomics: 16S rRNA sequencing, Metabolomics: UPLC/ToFMS	Enterobacteriaceae, cholate^b^, conjugated and sulphated bile acids^b^, taurine^b^, tryptophan^b^, adrenate^b^	Stercobilin^b^, acetyl-glutamic acid^b^, boldione^b^, estradiol^b^, androstenedione^b^, azelaic acid^b^	Jacobs et al. (2016)
Paediatric IBD (newly diagnosed, treatment naive)-CD (36); UC (20); IBD-U (13) vs. endoscopic non-IBD controls (29)	Stool, blood	Metabolomics: UPLC-MS/MS	Folate and pterine biosynthesis, purine metabolism^b^, amino acid metabolism, nicotinate and nicotinamide metabolism^b^, urea cycle, protein biosynthesis, bile acid biosynthesis^c^, sphingolipid metabolism^c^, ammonia recycling^c^, taurine metabolism^c^, oxidation of branched-chain FAs^c^, phospholipid metabolism^c^, glycerolipid metabolism^c^	L-tryptophan, kynurenic acid, aspartate, threonine, asparagine, cytosine, histidine^b^, taurine^b^	Kolho et al. (2017)
CD (208); UC (126); IBD-U (21); IBS (412) vs. HC (1,025)	Stool	Metagenomics: WGS	Sugar^a^ degradation, succinate fermentation, Aspartate and asparagine biosynthesis, arginine biosynthesis, lysine biosynthesis^b^, proline biosynthesis^c^, aromatic compounds degradation, saturated FA elongation^b^, specific fatty acid and lipid biosynthesis^b^, thiamin salvage^c^, *Bacteroides spp.,* Enterobacteriaceae^b^, Bacteroidaceae	Pyruvate and mixed acid fermentation^b^, general amino acid biosynthesis (see exceptions in previous column), tryptophan degradation^b^, valine degradation^b^, coenzyme A biosynthesis^b^, coenzyme B biosynthesis, specific fatty acid and lipid biosynthesis^b^, nucleotides and nucleosides biosynthesis, phosphopantothenate biosynthesis^b^, *Faecalibacterium prausnitzii* ^b^ *, Bifidobacterium longum* ^b^ *, Roseburia hominis,* Actinobacteria, Rikenellaceae, Akkermansiaceae, Firmicutes	Vich Vila et al. (2018)
Pediatric IBD (treatment naïve): CD (25); UC (22) vs. non-IBD (24)	Mucosal-luminal interface (MLI) biopsy	Metaproteomics: MS	DNA replication, recombination, and repair proteins, defence mechanism proteins (CRISPR/Cas), cell wall, membrane and envelope biogenesis proteins, amino acid transport and metabolism, mobilome, cysteine degradation, Proteobacteria, Verrucomicrobia, Ascomycota, Spirochetes*, F. prausnitzii* strain L2-6	Cysteine biosynthesis, *Bacteroides*	Zhang et al. (2019)
CD (68); UC (53) vs. non-IBD (34)	Stool	Metagenomics: WGS, Metabolomics: LC-MS	Sphingolipids, carboximic acids^a^, bile acids (cholate, chenodeoxycholate)^a^, organonitrogen compounds, cholesteryl esters, phenylacetamides^b^, phosphatidylcholines, α-amino acids, lactate	LCFAs, butyrate^d^, propionate^d^, secondary bile acids (lithocholate, deoxycholate) ^d^, flavonoids, indoles^a^ ^,^ ^b^, cinnamic acids^a^, triacylglycerols, tetrapyrroles^a^ ^,^ ^b^, triterpenoids, alkyl-phenylketones, brassinolides^a^ ^,^ ^b^, ergosterols^a^, quinolines^a^ ^,^ ^b^, vitamin D^a^, stigmastanes^a^, lactones, β-diketones^b^, cholesterols^a^, phenylbenzodioxanes, pantothenate (vitamin B5)	Franzosa et al. (2019)
CD (67); UC (38) vs. non-IBD (27)	Stool, colon biopsy, blood	Metagenomics: WGS, Metatranscriptomics, Metabolomics: LC-MS	Cholate^b^, chenodeoxycholate^b^, taurochenodeoxycholate^b^, C8 carnitine^b^, anti-ompc^b^, calprotectin^c^, adrenate, arachidonate, putrescine, taurine, *Escherichia coli, Klebsiella pneumoniae, Roseburia gnavus* ^b^	Deoxycholate, lithocholate, propionate, C16:0 LPE, adipate, C20:4 carnitine, 3'-O-methyladenosine, suberate, nicotinate (B3), pantothenate (B5), *F. prausnitzii*, *Alistipes finegoldii*, *Alistipes shahii*, *Alistipes putredinis, Subdoligranulum unclassified*	Lloyd-Price *et al.* (2019)
Treatment-naive UC (18) vs. HC (14)	Mucosal biopsy	Metabolomics: GC-ToFMS, UPLC-MS	Lysophospholipids^c^, acyl carnitines^c^, arachidonate^c^, asparagine^c^, citrulline^c^, dimethylarginine^c^, glutamyl-L-amino acids^c^, glutamate^c^, kynurenine^c^, L-valine^c^, L-isoleucine^c^, nicotinamide^c^	beta-alanine^c^, creatine^c^, eicosapentaenoate^c^, fructose^c^, glutaryl-carnitine^c^, glycerol-3-phosphate^c^, guanosine^c^, leucylglycine^c^, linoleate^c^, L-glutamine^c^, methylmalonyl carnitine^c^	Diab et al. (2019)

a Includes derivatives of the molecule class.

b Significantly different in CD only.

c Significantly different in UC only.

d Difference not statistically significant in this cohort.

Meta-omics studies have revealed that microbial dysbiosis in IBD and CRC goes beyond taxonomic imbalance. While there are discrepancies regarding the differential expression of specific proteins or metabolites between cohorts, the research collectively paints a picture of systemic dysregulation of multiple microbe-mediated compounds in disease. For example, amino acid and fatty acid metabolism are commonly dysregulated in IBD or CRC compared to healthy controls (Table 1), although the pattern of dysregulation is inconsistent. Other biochemical classes and pathways significantly dysregulated in a subset of studies include bile acids, vitamins B3 and B5, and sphingolipids. Interestingly, a similar pattern of metabolic dysregulation was apparent in the metabolome of a healthy CRC high-risk population (based on heritage and diet) compared to a healthy low-risk population (Ocvirk et al., 2020) (Table 1), suggesting that dysbiotic microbiomes are present before disease manifestation and contribute to CRC development. These shared patterns of dysregulation could indicate a shared etiology between IBD and CRC. The remainder of this review will summarize the biochemical significance of commonly dysregulated metabolites and pathways in the context of chronic inflammation and host immunity from *in vitro* and *in vivo* experimental research.

## Microbiome-Mediated Mechanisms and Impact on Homeostasis

### Amino Acid Metabolism and Polyamines

Many amino acids have an essential role in host immune signaling, and some are precursors for tumour-promoting compounds such as polyamines, which are capable of modulating systemic and mucosal adaptive immunity (Rooks and Garrett, 2016; Thomas et al., 2019). Genes involved in amino acid degradation were found to be enriched in a meta-analysis of CRC metagenomic profiles (Wirbel et al., 2019), and differential abundances of specific amino acids and related pathways are prevalent in IBD and CRC meta-omics studies (Table 1).

Both host and microbial cells utilize the essential amino acid tryptophan. Catabolism of tryptophan by macrophages leads to suppressed T cell proliferation *in vitro* (Munn et al., 1999). Tryptophan degradation has also been shown to modulate the differentiation of T cell subsets, affecting mucosal immunity and epithelial barrier integrity (Shapiro et al., 2014). Microbial metabolism of tryptophan can result in the production of kynurenines and indole-3-aldehyde, metabolites with immunomodulatory and anti-inflammatory effects including aryl hydrocarbon receptor activation promoting T_reg_ cell development and local IL-22 production (Zelante et al., 2013). Both tryptophan and kynurenic acid were found to be depleted in newly diagnosed, treatment-naive pediatric IBD patients (Kolho et al., 2017), whereas patients with remissive IBD displayed increased levels of tryptophan in stool compared to healthy controls (Jansson et al., 2009; Jacobs et al., 2016), supporting an immunoprotective role. Decreased tryptophan degradation in CD patients has also been predicted from deep sequencing of fecal metagenomes (Vich Vila et al., 2018). In contrast, kynurenine levels were elevated in mucosal biopsy samples of treatment-naive UC patients compared to healthy controls (Diab et al., 2019), as well as in stool from late-stage CRC patients (Yachida et al., 2019). Whether these discrepancies result from differing sample types or the contradictory effects of bioactive kynurenines (Rossi et al., 2019) is not yet understood.

Polyamines putrescine, spermidine, and spermine are derived from the amino acids arginine and ornithine in bacterial and mammalian cells (Gerner and Meyskens, 2004; Pegg, 2016; Rooks and Garrett, 2016). Other polyamines, such as trimethylamine (TMA), are synthesized by bacteria from the quaternary ammonium compounds choline and carnitine (Gerner and Meyskens, 2004; Thomas et al., 2019). Polyamines are essential metabolites for both the host and the microbiota. They are present at high levels in the GI tract, where they enable rapid turnover of intestinal epithelial cells and regulate specific eukaryotic gene expression among many other functions. For many decades now, polyamines have been implicated in tumorigenesis, including CRC (Gerner and Meyskens, 2004; Peng et al., 2021), due to their essential role in cell proliferation. Bacterial TMA production has also been linked to the consumption of red meat (a source of carnitine) and cardiovascular disease (Koeth et al., 2014).

In meta-omic CRC studies, levels of choline and choline-containing compounds (precursors of TMA) were found to be elevated in colorectal tumour biopsies relative to normal tissue from the same patients (Chan et al., 2009) and in stool from a high-risk group for CRC compared to a low-risk group (Ocvirk et al., 2020). Yachida et al. (2019) reported differentially abundant acetylated and diacetylated derivates of spermine, which have been previously proposed as tumour markers (Kawakita and Hiramatsu, 2006), at different stages of CRC. Of note, the polyamine putrescine and certain carnitine derivatives were reported to be differentially abundant in IBD compared to healthy controls (Diab et al., 2019; Lloyd-Price et al., 2019), supporting a role for polyamine metabolism in the etiology of IBD as well as CRC. Carnitine additionally serves as an organic compatible solute (osmoprotectant) for bacterial cells under osmotic stress (Meadows and Wargo, 2015). Hyperosmolarity is correlated to inflammation and serves as the primary inflammatory mechanism for DSS and other chemically-induced colitis models (Schwartz et al., 2009).

Levy et al. (2015) described altered amino acid and polyamine metabolism in the gut microbiome of NLRP6 inflammasome-deficient mice. Taurine—a bile acid conjugate—was depleted and shown to induce NLRP6-dependent IL-18 secretion by triggering intestinal inflammasome activation *in vitro*. The authors propose taurine as a microbiota-dependent positive inflammasome/IL-18 modulator and the polyamine spermine as an inflammasome/IL-18 suppressor. Epithelial IL-18 secretion leads to downstream antimicrobial peptide production, which in turn affects the intestinal microenvironment. The addition of taurine, histamine, or spermine to drinking water induced compositional changes in the gut microbiome of wild-type mice, but not in inflammasome-deficient mice nor anaerobic cultures, suggesting that the capacity of these molecules to alter the gut microbial balance depends on host signaling via NLRP6. When dysbiotic microbiota from inflammasome-deficient mice was transferred into a wild-type host, the authors observed dominance of the dysbiotic microbiota, leading to inflammasome suppression and reduced levels of colonic IL-18. These results highlight the complex interplay of host and microbial metabolism and the delicate inflammatory balance in the gut microenvironment. Patterns of dysregulation regarding amino acid metabolism in IBD (particularly in CD), are inconsistent with those observed in Irritable Bowel Syndrome (IBS) patients (Vich Vila et al., 2018), suggesting a disease-specific mechanism.

### Bile Acids

In healthy states, primary bile acids (BAs), including cholate and chenodeoxycholate, are synthesized from cholesterol in the liver and conjugated with glycine or taurine before excretion. The majority of primary BAs are reabsorbed in the ileum; however, a small proportion (approximately 5%) enter the colon where they are deconjugated and converted to secondary BAs, including deoxycholic acid and lithocholic acid, respectively, by the microbiota (Peng et al., 2021). Dominant members of the healthy stool microbiome (Human Microbiome Project Consortium, 2012), namely Firmicutes (including *Clostridium* spp., *Ruminococcus gnavus* and *Faecalibacterium prausnitzii*) and Bacteroidetes, are potent BA deconjugators and secondary BA transformation is attributed to metabolic activity by the genera *Bacteroides, Clostridium, Eubacterium, Lactobacillus*, and *Escherichia* (Duboc et al., 2013; Jia et al., 2018). In this regard, metabolism by the microbiome determines the composition of the BA pool in the gut. Bile acids in the intestine act primarily as detergents, facilitating the absorption of lipids and fat-soluble compounds. However, they can also act like hormones, regulating nutrient metabolism by activating specific nuclear and G protein-coupled receptors. They have also been shown to act as antimicrobial agents in the gut through their detergent activity (Shapiro et al., 2014; Ridlon et al., 2016). Ridlon et al. (2014, 2016) present in-depth reviews of bile acid metabolism and the gut microbiome.

Bile acids have been implicated in tumorigenesis, and particularly in CRC, through their capacity to induce oxidative stress and DNA damage at high concentrations (Bernstein et al., 2009; Ajouz et al., 2014; Jia et al., 2018). Increased levels of secondary BAs, specifically hydrophobic deoxycholic acid, have been correlated with the presence of colorectal adenomas (precursor lesions for CRC) and are capable of promoting intestinal tumorigenesis in experimental mouse models (Bernstein et al., 2009; Yachida et al., 2019; Peng et al., 2021). *Bilophilia wadsworthia*, whose growth is stimulated by the conjugated primary BA taurocholate, was enriched in patients with multiple polypoid adenomas and significantly correlated with the concentration of deoxycholic acid in stool (Yachida et al., 2019). Dietary fat intake positively influences secondary BA production, and Western-style diets with high fat intake have been implicated in the etiology of CRC (Bernstein et al., 2009; Ajouz et al., 2014). Metagenomic meta-analysis of the gut microbiome in CRC has indicated that genes for secondary BA conversion are consistently enriched in disease (Wirbel et al., 2019), and deoxycholic acid, along with cholate and chenodeoxycholate, were found to be enriched in a metabolomic analysis of a CRC high-risk cohort (Ocvirk et al., 2020). Another metabolomic analysis reported significantly elevated levels of glycine and taurine conjugates of cholate in early-stage CRC (Yachida et al., 2019). Metaproteomic analysis of CRC by Long et al. (2020) revealed elevated abundance of genera involved in BA metabolism and enrichment of DNA repair proteins and superoxide dismutases, indicating oxidative stress response (Jia et al., 2018; Long et al., 2020) (Table 1).

Despite their link to carcinogenesis, non-sulphated secondary BAs have been shown to exert anti-inflammatory effects *in vitro* (Duboc et al., 2013). Signaling via the main BA receptors, G protein-coupled receptor (GPCR) TGR5 and nuclear receptor farnesoid X receptor (FXR), leads to downstream inhibition of NF-κB mediated pro-inflammatory innate immune response (Shapiro et al., 2014). FXR activation has been shown to protect mice from induced colitis via the downregulation of pro-inflammatory cytokines (Gadaleta et al., 2011). It has also been demonstrated to play a role in maintaining intestinal epithelium integrity by preventing bacterial overgrowth (Inagaki et al., 2006). Conversely, inactivation of FXR and subsequent BA dysregulation has been associated with increased colon cell proliferation and tumorigenesis in mice fed a Western-style diet (Dermadi et al., 2017). Individual BAs have variable ability to activate FXR and TGR5; unconjugated BAs are considered to have a greater FXR activation potential than their conjugated counterparts, and secondary BAs (including conjugated forms) are potent activators of TGR5 (Jia et al., 2018). Meta-omics of IBD indicates that primary conjugated and unconjugated BAs are enriched, and secondary BAs are depleted in disease (Jansson et al., 2009; Jacobs et al., 2016; Franzosa et al., 2019; Lloyd-Price et al., 2019). This pattern has likewise been observed in IBD serum metabolomics (Duboc et al., 2013), indicating systemic disruption. These results support a disease model in which gut dysbiosis alters the BA pool in favour of primary BAs. The subsequent reduction in secondary BAs drives host immune signaling toward an inflammatory phenotype.

### Fatty Acids

Fatty acids, especially short-chain fatty acids (SCFAs), have received a great deal of attention in GI health. Colonocytes use SCFAs derived from gut microbial fermentation of dietary polysaccharides as an important source of energy (Shapiro et al., 2014; Rooks and Garrett, 2016; Hanus et al., 2021). These microbial metabolites can also contribute to host immunostasis through GPCR signaling and histone deacetylase (HDAC) inhibition, promoting an anti-inflammatory phenotype in the gut and strengthening the intestinal barrier (Shapiro et al., 2014; Rooks and Garrett, 2016). In particular, the SCFA butyrate and butyrate-producing bacteria, such as *Faecalibacterium prausnitzii* and *Roseburia hominis*, are considered to be beneficial, and their depletion is characteristic of IBD dysbiosis (Machiels et al., 2014; Patterson et al., 2017; Forbes et al., 2018; Alameddine et al., 2019). Butyrate is thought to contribute to intestinal health through a variety of mechanisms, including promotion of T_reg_ differentiation and macrophage polarization, inhibition of LPS-induced pro-inflammatory cytokine production, promotion of apoptosis in colonocytes, and strengthening intestinal epithelial cell (IEC) barrier function (Alameddine et al., 2019; Knox et al., 2019b; Silva et al., 2020; Hanus et al., 2021).

The protective role of SCFAs in CRC has also been suggested via their ability to epigenetically modulate tumour suppressor gene translation through HDAC inhibition and activation of GPCR signaling pathways resulting in colon adenoma and carcinoma cell apoptosis (Ou et al., 2013; Hanus et al., 2021; Peng et al., 2021). Although most of the meta-omic explorations of CRC discussed in this review did not report statistically significant differences in SCFAs between disease and controls, butyrate-producing species such as *Lachnospira multipara* and *Eubacterium eligens* were significantly (*p <* 0.005) depleted in CRC for one study, which also reported significant elevation of phenylpropionate in late-stage CRC, specifically (Yachida et al., 2019). Butyrate was found to be significantly less abundant in the high-risk CRC population described (Ocvirk et al., 2020) compared to the low-risk population. Similar results have been reported in another CRC-risk cohort study (Ou et al., 2013). The observation that butyrate and butyrate-producing species are depleted in both IBD and CRC suggests a metabolic link between diseases and possible mechanism for increased risk of CRC development among IBD patients via epigenetics.

The gut microbiota has also been implicated in the metabolism of a wide range of FAs, such as the polyunsaturated FAs arachidonic acid and linoleic acid, since conjugation and transformation of these metabolites was found to be dependent on the gut microbiota (Kishino et al., 2013). Linoleic and arachidonic acid are essential for the production of prostaglandins (PGs) and other eicosanoids, molecules that contribute to immune signaling and inflammation via cytokine production (Jansson et al., 2009). Increased production of PGs drives chronic intestinal inflammation and has been identified in the intestinal mucosa of IBD patients (Raab et al., 1995). However, there also exists a role for PGs in the maintenance and repair of intestinal epithelium (Wang et al., 2005). In CRC, arachidonic and linoleic acid were decreased in some cohorts (Chan et al., 2009; Phua et al., 2014), while others reported decreases in global lipid metabolism (Long et al., 2020).

Elevated levels of arachidonic acid were observed in IBD cohorts (Jansson et al., 2009; Diab et al., 2019; Lloyd-Price et al., 2019). A decrease in arachidonic acid metabolism and subsequent PG production, as observed in Jansson et al. (2009), may explain the accumulation of this FA in the IBD environment. Overall, the meta-omics data indicates broad disruptions in FA metabolism in disease, especially in IBD, where depletion of short-, medium-, and long-chain FAs has been consistently reported. Further experimental characterization of the complex interactions of FAs and their metabolites with host immune regulation in the context of gut inflammation may help to elucidate the precise role of FA metabolism in pathogenesis.

### Vitamins B3 & B5

B vitamins are key intermediates in essential cofactor metabolism and are indispensable for cellular life. These essential micronutrients are obtained from dietary sources, bacterial sources, or both. A deficiency of these vitamins from increased cellular demand or absorption defect leads to various physiological disruptions. Many, but not all, members of the gut microbiota are prototrophic for B vitamin synthesis (Peterson et al., 2020). It has been suggested that auxotrophic human gut bacteria rely on B vitamin sharing within the GI microenvironment for survival, as humanized gnotobiotic mice supported B vitamin auxotroph survival for at least 4 weeks regardless of dietary vitamin intake (Sharma et al., 2019).

Vitamin B3, also known as niacin or nicotinic acid, is a precursor of nicotinamide adenine dinucleotide (NAD), an essential cofactor involved in cellular redox reactions. Bacteria synthesize niacin from aspartic acid or tryptophan via the kynurenine pathway, ultimately resulting in NAD production. Human cells can also synthesize NAD *via* niacin-independent salvage pathways. In addition to its role in essential redox reactions, NAD plays a role in epigenetic enzyme regulation and genomic stability, as well as reactive oxygen species inhibition (Peterson et al., 2020). Administration of niacin disrupts NF-κB signaling, leading to suppression of inflammatory cytokines; its effects have been investigated in the context of several human diseases, including atherosclerosis, fatty liver disease, and Parkinson’s disease (Su et al., 2015; Peterson et al., 2020). Niacin is also known to affect fatty acid synthesis via NAD, and niacin or tryptophan deficiency can result in Pellagra, a disease whose symptoms include skin inflammation, diarrhea, and dementia. In mice, a diet supplemented with niacin and tryptophan was associated with increased expression of intestinal antimicrobial peptides and administration of this diet to colitis-susceptible mutant mice shifted the composition of the gut microbiota toward that of wild-type mice (Hashimoto et al., 2012). The receptor for niacin, GPR109A, is present on monocytes and macrophages and, notably, is also a receptor for the SCFA butyrate. Activation of GPR109A has been shown to suppress colonic inflammation and carcinogenesis via targeted T cell differentiation in mice and was deemed essential for butyrate-mediated IL-18 expression in the colonic epithelium (Singh et al., 2014).

Regarding meta-omics, niacin was reported to be decreased in CRC compared to healthy controls (Phua et al., 2014). The low-risk CRC cohort revealed elevated levels of niacin compared to the high-risk group (Ocvirk et al., 2020). In the same study, nicotinamide, a vitamer of niacin with anti-inflammatory properties (Peterson et al., 2020), and other niacin metabolites were enriched in the high-CRC-risk population (Ocvirk et al., 2020). Nicotinamide is suggested to play a role in cancer chemoprevention via enhanced DNA repair and suppression of pro-inflammatory mediators (Nikas et al., 2020). Loss of niacin-producing organisms in the gut could thus decrease the bioavailability of niacin to IECs, promoting inflammation and carcinogenesis. In IBD, vitamin B3 and associated metabolites appear to be differentially expressed in disease (Table 1). However, the pattern of dysregulation is inconsistent between cohorts (Kolho et al., 2017; Diab et al., 2019; Lloyd-Price et al., 2019). Differences in the type of biological sample(s) used, as well as cohort design, likely contribute to these inconsistencies. As already discussed, the stool and mucosal GI metabolic profiles can provide distinct contextual information (e.g., increased metabolite utilization or reabsorption in the GI tract mucosa may result in decreased stool concentrations or vice versa). The interpretation of these results is additionally challenging since niacin/NAD metabolism affects many cellular processes.

Vitamin B5, pantothenic acid, is essential for coenzyme A (CoA) synthesis and is abundant in a large variety of foods, so deficiency is rare (Peterson et al., 2020). The sodium-dependent multivitamin transporter (SMVT) facilitates the uptake of vitamins B5 and B7. It has been demonstrated to play a role in gut permeability in mice (Sabui et al., 2018), implicating one or both of these vitamins in gut homeostasis. While there is a lack of evidence for the pantothenic acid disruption in CRC meta-omics, three relatively large-scale IBD studies reported significantly depleted vitamin B5 in disease compared to healthy controls (Vich Vila et al., 2018; Franzosa et al., 2019; Lloyd-Price et al., 2019) (Table 1). Depletion of pantothenic acid in the gut may be a symptom of IBD-related dysbiosis and the loss of B5-producing organisms; however, further investigation into the role of vitamin B5 in IBD is needed to understand the cause and effects of this perturbation.

### Sphingolipids

Sphingolipids such as sphingomyelin (SM) and glycosphingolipids (GSLs) are essential structural components of IEC membranes. In addition to a direct role in maintaining epithelial barrier integrity, sphingolipids and related metabolites have also been demonstrated to participate in immune signaling and modulation of inflammation (An et al., 2014; Abdel Hadi et al., 2016). In a mouse model, early-life exposure to microbial sphingolipids was shown to reduce invariant natural killer T (iNKT) cell proliferation and protect the adult host from iNKT cell-mediated colitis (An et al., 2014). Sphingolipid metabolism is complex and is reviewed in detail elsewhere (Abdel Hadi et al., 2016).

Sphingosine and ceramide are sphingolipids that, when accumulated on the surface of IECs, increase epithelial permeability and disrupt normal barrier function; ceramide was additionally found to induce either pro- or anti-inflammatory effects depending on its enzymatic origin (Abdel Hadi et al., 2016). The production of PGs from arachidonic acid, as previously discussed, can also be modulated by sphingolipid composition in cellular membranes (Nakamura and Murayama, 2014). Meta-omics of IBD reveals enrichment of sphingolipids (specifically, ceramide and sphingomyelin) and sphingolipid metabolism in disease (Kolho et al., 2017; Franzosa et al., 2019) (Table 1), supporting their role as pro-inflammatory mediators. No significant differences in sphingolipid metabolism were reported for CRC (Table 1). However, disruptions in arachidonic acid metabolism and PG production (Chan et al., 2009) may indicate undetected upstream changes in sphingolipid composition. Experimental evidence has tied sphingolipid receptor expression to tumour suppression in CRC (Petti et al., 2020). Due to their role in intestinal epithelial barrier maintenance and their varied capability for immune signaling, further characterization of sphingolipids in intestinal disease is warranted. Notably, further experimentation with early-life exposure to microbial sphingolipids and the resulting effects on host immune development could have implications related to the hygiene hypothesis (see below) and IBD development.

## Summary

Technological advancements in recent decades have enabled extensive characterization of the human gut microbiome via meta-omics techniques. IBD, in particular, is commonly modelled in microbiome research, and the association with CRC is thought to indicate some degree of shared etiology between the two diseases. Genetic, environmental, and immunologic factors are thought to contribute to disease development; however, the mechanisms by which they do so are complex and not fully understood (Sobhani et al., 2011; Carroll et al., 2019). The intestinal microbiota have been implicated in the pathogenesis of both IBD and CRC. In the absence of a directly causative agent, the microbial community within the gut is being investigated as a mutualistic pseudo-organ capable of influencing homeostasis in its host.

It has been suggested that increased urbanization and hygienic behaviours (*e.g.,* household use of disinfectants) results in reduced colonization by commensal organisms in early life and that this lack of exposure directly affects immune system development, resulting in a predisposition to aberrant immune responses—*i.e.,* IBD—later in life (Weinstock and Elliott, 2009). Much support for this “hygiene hypothesis” comes from experimental studies regarding helminth and parasite colonization; however, these organisms are not typically captured in microbiome studies. Given the impact of the early-life environment on individual microbiome development and subsequent health outcomes (Nielsen et al., 2020; Boutin et al., 2021), the role of commensal bacteria in immune development and sensitization should not be ignored. Meta-omic cohort studies can provide insights into the mechanisms of aberrant immune response in disease and identify key metabolites whose role in immune development warrants further study.

An advantage of functional meta-omics is the potential to discover molecular biomarkers of disease that can aid in non-invasive screening and diagnosis, or in the case of IBD, distinguishing component diseases from one another. Meta-omic characterization of IBD and CRC in human cohorts has revealed similar patterns of metabolic dysregulation that implicate a disruption in host-microbiota cross-talk leading to aberrant immune response and inflammation. Dysregulated metabolism of amino acids, polyamines, bile acids, fatty acids, and B vitamins has been reported in both IBD and CRC cohorts, supporting a degree of shared microbiome-mediated etiology between the diseases. Several of these metabolites directly or indirectly affect NF-κB activation, a key transcription factor within the intestinal tumour microenvironment (Schwitalla et al., 2013). Protective microbially-mediated compounds such as tryptophan and butyrate have been shown to impact T_reg_ cell proliferation, and their relative decrease in disease not only reflects the loss of beneficial organisms, but also potentially drives autoinflammation through imbalanced T cell differentiation (Dominguez-Villar and Hafler, 2018). Although IBD are risk factors for CRC, not all IBD patients develop malignancies. Changes in the GI microbiome have been associated with cancer development in animal studies; however, the precise role of the microbiome in mediating carcinogenesis has yet to be discovered.

The human genetic landscape is one of many health determinants that can influence the development of individual gut microbiomes (Goodrich et al., 2014). Alternatively, studies in mice have revealed that dysbiotic fecal microbiota from a genetically susceptible (*e.g.,* Nod2 or Asc deficient) host can be dominantly transferred to healthy, wild-type recipients and is sufficient to increase sensitivity to DSS-induced colitis and tumorigenesis (Couturier-Maillard et al., 2013; Levy et al., 2015), indicating that dysbiotic communities have the capacity to increase disease risk in wild-type recipients *via* molecular signaling (*e.g.,* cytokine modulation) under these experimental conditions. These data support a model whereby inflammation and disease susceptibility depend on critical communication between the microbiome and the immune system of the host. Dysbiosis may be driven by a multitude of factors, including genetic susceptibility, diet, antibiotic usage, smoking status and other environmental exposures, which could have individual as well as cumulative effects on the gut microenvironment, including epithelial barrier integrity and inflammation. Despite these diverse influences, there appears to be some degree of shared microbially-mediated metabolic dysregulation in IBD and CRC, supporting a common etiology related to the GI microbiome. The discovery of significant metabolic differences between the microbiomes of healthy individuals in either high- or low-risk CRC cohorts suggests a model of dysbiotic metabolism in asymptomatic individuals leading to enhanced risk of immune imbalance and symptomatic disease.

Due to a high degree of variability in meta-omics data, systematic meta-analyses can be particularly useful in revealing disease-specific microbiome signatures across cohorts (Wirbel et al., 2019). Future research directions include longitudinal metabolic characterization of the IBD microbiome, including quantification of specific metabolites discussed here, toward development of CRC in order to identify why some IBD patients develop CRC and others do not. The role of microbial metabolites in infant immune system development may also provide valuable insights into immune modulation and disease susceptibility. Additionally, data produced from downstream meta-omics techniques (*i.e.,* metaproteomics and metabolomics) can be used to confirm or contradict predicted functional activity from the plethora of available metataxonomics, metagenomics, or metatranscriptomics research. Even if consistent changes in gut meta-omics are found that are predictive of persons with IBD who develop CRC there remains the issue of determining cause and effect. Future studies will benefit from large cohort sizes, detailed metadata, considerate sample collection and storage techniques, and robust statistical approaches in order to address the many challenges associated with meta-omics research. However, there are studies that show that colon neoplasia is most likely to develop in the setting of active inflammation and is unlikely to develop in the absence of inflammation (Rubin et al., 2013; Shaffer et al., 2021; Yvellez et al., 2021). The association of meta-omics changes with other non-IBD chronic immune mediated inflammatory diseases suggests that the intestinal microbial milieu may drive systemic inflammation (Forbes et al., 2018). An evolving paradigm of gut microbial changes driving inflammation and the knowledge that gut inflammation can drive neoplasia development, makes it realistic to consider that microbial changes may underlie the ultimate development of neoplasia in persons with IBD and identifying these changes early on can be a mechanism to interrupt the inflammation-neoplasia paradigm in IBD. As the field continues to expand, overcoming barriers to clinical implementation of meta-omics will pave the way for personalized approaches to diagnosis and screening.

## References

[B1] Abdel Hadi L. Di Vito C. Riboni L. (2016). Fostering Inflammatory Bowel Disease: Sphingolipid Strategies to Join Forces. Mediators Inflamm. 13, 1. 10.1155/2016/3827684 PMC4736332 26880864

[B3] Ajouz H. Mukherji D. Shamseddine A. (2014). Secondary Bile Acids: an Underrecognized Cause of colon Cancer. World J. Surg. Onc 12, 1–5. 10.1186/1477-7819-12-164 PMC4041630 24884764

[B4] Akimoto N. Ugai T. Zhong R. Hamada T. Fujiyoshi K. Giannakis M. (2020). Rising Incidence of Early-Onset Colorectal Cancer - a Call to Action. Nat. Rev. Clin. Oncol. 18, 230–243. 10.1038/s41571-020-00445-1 33219329 PMC7994182

[B5] Alameddine J. Godefroy E. Papargyris L. Sarrabayrouse G. Tabiasco J. Bridonneau C. (2019). Faecalibacterium Prausnitzii Skews Human DC to Prime IL10-Producing T Cells through TLR2/6/JNK Signaling and IL-10, IL-27, CD39, and Ido-1 Induction. Front. Immunol. 10. 10.3389/fimmu.2019.00143 PMC6373781 30787928

[B6] Alberti A. Belser C. Engelen S. Bertrand L. Orvain C. Brinas L. (2014). Comparison of Library Preparation Methods Reveals Their Impact on Interpretation of Metatranscriptomic Data. BMC Genomics 15, 912. 10.1186/1471-2164-15-912 25331572 PMC4213505

[B7] An D. Oh S. F. Olszak T. Neves J. F. Avci F. Y. Erturk-Hasdemir D. (2014). Sphingolipids from a Symbiotic Microbe Regulate Homeostasis of Host Intestinal Natural Killer T Cells. Cell 156, 123–133. 10.1016/j.cell.2013.11.042 24439373 PMC3909465

[B8] Atarashi K. Tanoue T. Shima T. Imaoka A. Kuwahara T. Momose Y. (2011). Induction of Colonic Regulatory T Cells by Indigenous Clostridium Species. Science 331, 337–341. 10.1126/science.1198469 21205640 PMC3969237

[B9] Becattini S. Sorbara M. T. Kim S. G. Littmann E. L. Dong Q. Walsh G. (2021). Rapid Transcriptional and Metabolic Adaptation of Intestinal Microbes to Host Immune Activation. Cell Host & Microbe 29, 378–393. 10.1016/j.chom.2021.01.003 33539766 PMC7954923

[B10] Bernstein H. Bernstein C. Payne C. M. Dvorak K. (2009). Bile Acids as Endogenous Etiologic Agents in Gastrointestinal Cancer. Wjg 15, 3329–3340. 10.3748/wjg.15.3329 19610133 PMC2712893

[B11] Boutin R. C. Petersen C. Woodward S. E. Serapio-Palacios A. Bozorgmehr T. Loo R. (2021). Bacterial-fungal Interactions in the Neonatal Gut Influence Asthma Outcomes Later in Life. eLife 10. 10.7554/eLife.67740 PMC8075585 33876729

[B12] Carroll M. W. Kuenzig M. E. Mack D. R. Otley A. R. Griffiths A. M. Kaplan G. G. (2019). The Impact of Inflammatory Bowel Disease in Canada 2018: Children and Adolescents with IBD. J. Can. Assoc. Gastroenterol. 2, S49–S67. 10.1093/jcag/gwy056 31294385 PMC6512244

[B13] Chan E. C. Y. Koh P. K. Mal M. Cheah P. Y. Eu K. W. Backshall A. (2009). Metabolic Profiling of Human Colorectal Cancer Using High-Resolution Magic Angle Spinning Nuclear Magnetic Resonance (HR-MAS NMR) Spectroscopy and Gas Chromatography Mass Spectrometry (GC/MS). J. Proteome Res. 8, 352–361. 10.1021/pr8006232 19063642

[B14] Couturier-Maillard A. Secher T. Rehman A. Normand S. De Arcangelis A. Haesler R. (2013). NOD2-mediated Dysbiosis Predisposes Mice to Transmissible Colitis and Colorectal Cancer. J. Clin. Invest. 123, 700–711. 10.1172/JCI62236 23281400 PMC3561825

[B15] Dermadi D. Valo S. Ollila S. Soliymani R. Sipari N. Pussila M. (2017). Western Diet Deregulates Bile Acid Homeostasis, Cell Proliferation, and Tumorigenesis in Colon. Cancer Res. 77, 3352–3363. 10.1158/0008-5472.CAN-16-2860 28416481

[B16] Diab J. Hansen T. Goll R. Stenlund H. Jensen E. Moritz T. (2019). Mucosal Metabolomic Profiling and Pathway Analysis Reveal the Metabolic Signature of Ulcerative Colitis. Metabolites 9, 291. 10.3390/metabo9120291 PMC6950742 31783598

[B17] Dominguez-Villar M. Hafler D. A. (2018). Regulatory T Cells in Autoimmune Disease. Nat. Immunol. 19, 665–673. 10.1038/s41590-018-0120-4 29925983 PMC7882196

[B18] Duboc H. Rajca S. Rainteau D. Benarous D. Maubert M.-A. Quervain E. (2013). Connecting Dysbiosis, Bile-Acid Dysmetabolism and Gut Inflammation in Inflammatory Bowel Diseases. Gut 62, 531–539. 10.1136/gutjnl-2012-302578 22993202

[B19] El-Matary W. Bernstein C. N. (2020). Cancer Risk in Pediatric-Onset Inflammatory Bowel Disease. Front. Pediatr. 8. 10.3389/fped.2020.00400 PMC7396532 32903330

[B20] Forbes J. D. Chen C.-y. Knox N. C. Marrie R.-A. El-Gabalawy H. de Kievit T. (2018). A Comparative Study of the Gut Microbiota in Immune-Mediated Inflammatory Diseases-Does a Common Dysbiosis Exist? Microbiome 6, 221. 10.1186/s40168-018-0603-4 30545401 PMC6292067

[B21] Franzosa E. A. Sirota-Madi A. Avila-Pacheco J. Fornelos N. Haiser H. J. Reinker S. (2019). Gut Microbiome Structure and Metabolic Activity in Inflammatory Bowel Disease. Nat. Microbiol. 4, 293–305. 10.1038/s41564-018-0306-4 30531976 PMC6342642

[B22] Gadaleta R. M. van Erpecum K. J. Oldenburg B. Willemsen E. C. L. Renooij W. Murzilli S. (2011). Farnesoid X Receptor Activation Inhibits Inflammation and Preserves the Intestinal Barrier in Inflammatory Bowel Disease. Gut 60, 463–472. 10.1136/gut.2010.212159 21242261

[B23] Gausman V. Dornblaser D. Anand S. Hayes R. B. O'Connell K. Du M. (2020). Risk Factors Associated with Early-Onset Colorectal Cancer. Clin. Gastroenterol. Hepatol. 18, 2752–2759. e2. 10.1016/j.cgh.2019.10.009 31622737 PMC7153971

[B24] Gerner E. W. Meyskens F. L. (2004). Polyamines and Cancer: Old Molecules, New Understanding. Nat. Rev. Cancer 4, 781–792. 10.1038/nrc1454 15510159

[B25] Gevers D. Kugathasan S. Denson L. A. Vázquez-Baeza Y. Van Treuren W. Ren B. (2014). The Treatment-Naive Microbiome in New-Onset Crohn's Disease. Cell Host & Microbe 15, 382–392. 10.1016/j.chom.2014.02.005 24629344 PMC4059512

[B26] Glória L. Cravo M. Pinto A. de Sousa L. S. Chaves P. Leitão C. N. (1996). DNA Hypomethylation and Proliferative Activity Are Increased in the Rectal Mucosa of Patients with Long-Standing Ulcerative Colitis. Cancer 78, 2300–2306. 10.1002/(sici)1097-0142(19961201)78:11<2300:aid-cncr5>3.0.co;2-q 8940998

[B27] Goodrich J. K. Waters J. L. Poole A. C. Sutter J. L. Koren O. Blekhman R. (2014). Human Genetics Shape the Gut Microbiome. Cell 159, 789–799. 10.1016/j.cell.2014.09.053 25417156 PMC4255478

[B28] Hall A. B. Yassour M. Sauk J. Garner A. Jiang X. Arthur T. (2017). A Novel Ruminococcus Gnavus Clade Enriched in Inflammatory Bowel Disease Patients. Genome Med. 9, 103. 10.1186/s13073-017-0490-5 29183332 PMC5704459

[B29] Hanus M. Parada-Venegas D. Landskron G. Wielandt A. M. Hurtado C. Alvarez K. (2021). Immune System, Microbiota, and Microbial Metabolites: The Unresolved Triad in Colorectal Cancer Microenvironment. Front. Immunol. 12. 10.3389/fimmu.2021.612826 PMC8033001 33841394

[B30] Hashimoto T. Perlot T. Rehman A. Trichereau J. Ishiguro H. Paolino M. (2012). ACE2 Links Amino Acid Malnutrition to Microbial Ecology and Intestinal Inflammation. Nature 487, 477–481. 10.1038/nature11228 22837003 PMC7095315

[B31] Himmel M. E. Hardenberg G. Piccirillo C. A. Steiner T. S. Levings M. K. (2008). The Role of T-Regulatory Cells and Toll-like Receptors in the Pathogenesis of Human Inflammatory Bowel Disease. Immunology 125, 145–153. 10.1111/j.1365-2567.2008.02939.x 18798918 PMC2561137

[B32] Human Microbiome Project Consortium (2012). Structure, Function and Diversity of the Healthy Human Microbiome. Nature 486, 207–214. 10.1038/nature11234 22699609 PMC3564958

[B33] Inagaki T. Moschetta A. Lee Y.-K. Peng L. Zhao G. Downes M. (2006). Regulation of Antibacterial Defense in the Small Intestine by the Nuclear Bile Acid Receptor. Proc. Natl. Acad. Sci. 103, 3920–3925. 10.1073/pnas.0509592103 16473946 PMC1450165

[B34] Jacobs J. P. Goudarzi M. Singh N. Tong M. McHardy I. H. Ruegger P. (2016). A Disease-Associated Microbial and Metabolomics State in Relatives of Pediatric Inflammatory Bowel Disease Patients. Cell Mol. Gastroenterol. Hepatol. 2, 750–766. 10.1016/j.jcmgh.2016.06.004 28174747 PMC5247316

[B35] Jansson J. Willing B. Lucio M. Fekete A. Dicksved J. Halfvarson J. (2009). Metabolomics Reveals Metabolic Biomarkers of Crohn's Disease. PLoS ONE 4, e6386. 10.1371/journal.pone.0006386 19636438 PMC2713417

[B36] Jia W. Xie G. Jia W. (2018). Bile Acid-Microbiota Crosstalk in Gastrointestinal Inflammation and Carcinogenesis. Nat. Rev. Gastroenterol. Hepatol. 15, 111–128. 10.1038/nrgastro.2017.119 29018272 PMC5899973

[B37] Jostins L. Ripke S. Weersma R. K. Duerr R. H. McGovern D. P. Hui K. Y. (2012). Host-microbe Interactions Have Shaped the Genetic Architecture of Inflammatory Bowel Disease. Nature 491, 119–124. 10.1038/nature11582 23128233 PMC3491803

[B38] Kaplan G. G. Bernstein C. N. Coward S. Bitton A. Murthy S. K. Nguyen G. C. (2019). The Impact of Inflammatory Bowel Disease in Canada 2018: Epidemiology. J. Can. Assoc. Gastroenterol. 2, S6–S16. 10.1093/jcag/gwy054 31294381 PMC6512243

[B39] Kawakita M. Hiramatsu K. (2006). Diacetylated Derivatives of Spermine and Spermidine as Novel Promising Tumor Markers. J. Biochem. 139, 315–322. 10.1093/jb/mvj068 16567395

[B40] Kishino S. Takeuchi M. Park S.-B. Hirata A. Kitamura N. Kunisawa J. (2013). Polyunsaturated Fatty Acid Saturation by Gut Lactic Acid Bacteria Affecting Host Lipid Composition. Proc. Natl. Acad. Sci. 110, 17808–17813. 10.1073/pnas.1312937110 24127592 PMC3816446

[B41] Knox N. C. Forbes J. D. Peterson C.-L. Van Domselaar G. Bernstein C. N. (2019b). The Gut Microbiome in Inflammatory Bowel Disease: Lessons Learned from Other Immune-Mediated Inflammatory Diseases. Am. J. Gastroenterol. 114, 1051–1070. 10.14309/ajg.0000000000000305 31232832

[B42] Knox N. C. Forbes J. D. Van Domselaar G. Bernstein C. N. (2019a). The Gut Microbiome as a Target for IBD Treatment: Are We There yet? Curr. Treat. Options. Gastro 17, 115–126. 10.1007/s11938-019-00221-w 30661163

[B43] Koeth R. A. Levison B. S. Culley M. K. Buffa J. A. Wang Z. Gregory J. C. (2014). γ-Butyrobetaine Is a Proatherogenic Intermediate in Gut Microbial Metabolism of L -Carnitine to TMAO. Cel Metab. 20, 799–812. 10.1016/j.cmet.2014.10.006 PMC4255476 25440057

[B44] Kolho K.-L. Pessia A. Jaakkola T. de Vos W. M. Velagapudi V. (2017). Faecal and Serum Metabolomics in Paediatric Inflammatory Bowel Disease. Eccojc 11, jjw158–334. 10.1093/ecco-jcc/jjw158 27609529

[B45] Krassowski M. Das V. Sahu S. K. Misra B. B. (2020). State of the Field in Multi-Omics Research: From Computational Needs to Data Mining and Sharing. Front. Genet. 11, 610798. 10.3389/fgene.2020.610798 33362867 PMC7758509

[B46] Levy M. Thaiss C. A. Zeevi D. Dohnalová L. Zilberman-Schapira G. Mahdi J. A. (2015). Microbiota-Modulated Metabolites Shape the Intestinal Microenvironment by Regulating NLRP6 Inflammasome Signaling. Cell 163, 1428–1443. 10.1016/j.cell.2015.10.048 26638072 PMC5665753

[B47] Lloyd-Price J. Arze C. Arze C. Ananthakrishnan A. N. Schirmer M. Avila-Pacheco J. (2019). Multi-omics of the Gut Microbial Ecosystem in Inflammatory Bowel Diseases. Nature 569, 655–662. 10.1038/s41586-019-1237-9 31142855 PMC6650278

[B48] Long S. Yang Y. Shen C. Wang Y. Deng A. Qin Q. (2020). Metaproteomics Characterizes Human Gut Microbiome Function in Colorectal Cancer. NPJ Biofilms Microbiomes 6. 10.1038/s41522-020-0123-4 PMC7093434 32210237

[B49] Lorzadeh A. Romero-Wolf M. Goel A. Jadhav U. (2021). Epigenetic Regulation of Intestinal Stem Cells and Disease: A Balancing Act of DNA and Histone Methylation. Gastroenterology 160, 2267–2282. 10.1053/j.gastro.2021.03.036 33775639 PMC8169626

[B50] Machiels K. Joossens M. Sabino J. De Preter V. Arijs I. Eeckhaut V. (2014). A Decrease of the Butyrate-Producing speciesRoseburia hominisandFaecalibacterium Prausnitziidefines Dysbiosis in Patients with Ulcerative Colitis. Gut 63, 1275–1283. 10.1136/gutjnl-2013-304833 24021287

[B51] Meadows J. A. Wargo M. J. (2015). Carnitine in Bacterial Physiology and Metabolism. Microbiology 161, 1161–1174. 10.1099/mic.0.000080 25787873 PMC4635513

[B52] Méndez-García C. Barbas C. Ferrer M. Rojo D. (2018). Complementary Methodologies to Investigate Human Gut Microbiota in Host Health, Working towards Integrative Systems Biology. J. Bacteriol. 200. 10.1128/JB.00376-17 PMC5763049 28874411

[B53] Molodecky N. A. Soon I. S. Rabi D. M. Ghali W. A. Ferris M. Chernoff G. (2012). Increasing Incidence and Prevalence of the Inflammatory Bowel Diseases with Time, Based on Systematic Review. Gastroenterology 142, 46–54. e42. 10.1053/j.gastro.2011.10.001 22001864

[B54] Munn D. H. Shafizadeh E. Attwood J. T. Bondarev I. Pashine A. Mellor A. L. (1999). Inhibition of T Cell Proliferation by Macrophage Tryptophan Catabolism. J. Exp. Med. 189, 1363–1372. 10.1084/jem.189.9.1363 10224276 PMC2193062

[B55] Nakamura H. Murayama T. (2014). The Role of Sphingolipids in Arachidonic Acid Metabolism. J. Pharmacol. Sci. 124, 307–312. 10.1254/jphs.13r18cp 24599139

[B56] Neuberger-Castillo L. Ammerlaan W. Betsou F. (2021). Fitness for Purpose of Stabilized Stool Samples for Bile Acid Metabolite Analyses. Sci. Rep. 11, 7904. 10.1038/s41598-021-86784-0 33846363 PMC8042040

[B57] Nielsen C. C. Gascon M. Osornio-Vargas A. R. Shier C. Guttman D. S. Becker A. B. (2020). Natural Environments in the Urban Context and Gut Microbiota in Infants. Environ. Int. 142, 105881. 10.1016/j.envint.2020.105881 32610248

[B58] Nikas I. P. Paschou S. A. Ryu H. S. (2020). The Role of Nicotinamide in Cancer Chemoprevention and Therapy. Biomolecules 10, 477. 10.3390/biom10030477 PMC7175378 32245130

[B59] Ocvirk S. Wilson A. S. Posma J. M. Li J. V. Koller K. R. Day G. M. (2020). A Prospective Cohort Analysis of Gut Microbial Co-metabolism in Alaska Native and Rural African People at High and Low Risk of Colorectal Cancer. Am. J. Clin. Nutr. 111, 406–419. 10.1093/ajcn/nqz301 31851298 PMC6997097

[B60] Ou J. Carbonero F. Zoetendal E. G. DeLany J. P. Wang M. Newton K. (2013). Diet, Microbiota, and Microbial Metabolites in colon Cancer Risk in Rural Africans and African Americans. Am. J. Clin. Nutr. 98, 111–120. 10.3945/ajcn.112.056689 23719549 PMC3683814

[B61] Patterson A. M. Mulder I. E. Travis A. J. Lan A. Cerf-Bensussan N. Gaboriau-Routhiau V. (2017). Human Gut Symbiont Roseburia Hominis Promotes and Regulates Innate Immunity. Front. Immunol. 8. 10.3389/fimmu.2017.01166 PMC5622956 29018440

[B62] Pegg A. E. (2016). Functions of Polyamines in Mammals. J. Biol. Chem. 291, 14904–14912. 10.1074/jbc.R116.731661 27268251 PMC4946908

[B63] Peloquin J. M. Nguyen D. D. (2013). The Microbiota and Inflammatory Bowel Disease: Insights from Animal Models. Anaerobe 24, 102–106. 10.1016/j.anaerobe.2013.04.006 23603043 PMC3766478

[B64] Peng Y. Nie Y. Yu J. Wong C. C. (2021). Microbial Metabolites in Colorectal Cancer: Basic and Clinical Implications. Metabolites 11, 159. 10.3390/metabo11030159 33802045 PMC8001357

[B65] Peterson C. T. Rodionov D. A. Osterman A. L. Peterson S. N. (2020). B Vitamins and Their Role in Immune Regulation and Cancer. Nutrients 12, 3380. 10.3390/nu12113380 PMC7693142 33158037

[B66] Petti L. Rizzo G. Rubbino F. Elangovan S. Colombo P. Restelli S. (2020). Unveiling Role of Sphingosine-1-Phosphate Receptor 2 as a Brake of Epithelial Stem Cell Proliferation and a Tumor Suppressor in Colorectal Cancer. J. Exp. Clin. Cancer Res. 39, 253. 10.1186/s13046-020-01740-6 33225975 PMC7682101

[B67] Phua L. C. Chue X. P. Koh P. K. Cheah P. Y. Ho H. K. Chan E. C. Y. (2014). Non-invasive Fecal Metabonomic Detection of Colorectal Cancer. Cancer Biol. Ther. 15, 389–397. 10.4161/cbt.27625 24424155 PMC3979816

[B68] Quince C. Walker A. W. Simpson J. T. Loman N. J. Segata N. (2017). Shotgun Metagenomics, from Sampling to Analysis. Nat. Biotechnol. 35, 833–844. 10.1038/nbt.3935 28898207

[B69] Raab Y. Sundberg C. Hällgren R. Knutson L. Gerdin B. (1995). Mucosal Synthesis and Release of Prostaglandin E2 from Activated Eosinophils and Macrophages in Ulcerative Colitis. Am. J. Gastroenterol. 90, 614–620. 7717321

[B70] Reck M. Tomasch J. Tomasch J. Deng Z. Jarek M. Husemann P. (2015). Stool Metatranscriptomics: A Technical Guideline for mRNA Stabilisation and Isolation. BMC Genomics 16. 10.1186/s12864-015-1694-y PMC4490624 26140923

[B71] Ridlon J. M. Harris S. C. Bhowmik S. Kang D.-J. Hylemon P. B. (2016). Consequences of Bile Salt Biotransformations by Intestinal Bacteria. Gut Microbes 7, 22–39. 10.1080/19490976.2015.1127483 26939849 PMC4856454

[B72] Ridlon J. M. Kang D. J. Hylemon P. B. Bajaj J. S. (2014). Bile Acids and the Gut Microbiome. Curr. Opin. Gastroenterol. 30, 332–338. 10.1097/MOG.0000000000000057 24625896 PMC4215539

[B73] Rooks M. G. Garrett W. S. (2016). Gut Microbiota, Metabolites and Host Immunity. Nat. Rev. Immunol. 16, 341–352. 10.1038/nri.2016.42 27231050 PMC5541232

[B74] Rossi F. Miggiano R. Ferraris D. M. Rizzi M. (2019). The Synthesis of Kynurenic Acid in Mammals: An Updated Kynurenine Aminotransferase Structural KATalogue. Front. Mol. Biosci. 6. 10.3389/fmolb.2019.00007 PMC6400995 30873412

[B75] Rubin D. T. Huo D. Kinnucan J. A. Sedrak M. S. McCullom N. E. Bunnag A. P. (2013). Inflammation Is an Independent Risk Factor for Colonic Neoplasia in Patients with Ulcerative Colitis: a Case-Control Study. Clin. Gastroenterol. Hepatol. 11, 1601e1–16084. 10.1016/j.cgh.2013.06.023 23872237 PMC3840031

[B76] Sabui S. Kapadia R. Ghosal A. Schneider M. Lambrecht N. W. G. Said H. M. (2018). Biotin and Pantothenic Acid Oversupplementation to Conditional SLC5A6 KO Mice Prevents the Development of Intestinal Mucosal Abnormalities and Growth Defects. Am. J. Physiology-Cell Physiol. 315, C73–C79. 10.1152/ajpcell.00319.2017 PMC6087731 29669219

[B77] Schirmer M. Franzosa E. A. Lloyd-Price J. McIver L. J. Schwager R. Poon T. W. (2018). Dynamics of Metatranscription in the Inflammatory Bowel Disease Gut Microbiome. Nat. Microbiol. 3, 337–346. 10.1038/s41564-017-0089-z 29311644 PMC6131705

[B78] Schwartz L. Guais A. Pooya M. Abolhassani M. (2009). Is Inflammation a Consequence of Extracellular Hyperosmolarity? J. Inflamm. 6, 21. 10.1186/1476-9255-6-21 PMC2709204 19549308

[B79] Schwitalla S. Fingerle A. A. Cammareri P. Nebelsiek T. Göktuna S. I. Ziegler P. K. (2013). Intestinal Tumorigenesis Initiated by Dedifferentiation and Acquisition of Stem-cell-like Properties. Cell 152, 25–38. 10.1016/j.cell.2012.12.012 23273993

[B80] Sellon R. K. Tonkonogy S. Schultz M. Dieleman L. A. Grenther W. Balish E. (1998). Resident Enteric Bacteria Are Necessary for Development of Spontaneous Colitis and Immune System Activation in Interleukin-10-Deficient Mice. Infect. Immun. 66, 5224–5231. 10.1128/IAI.66.11.5224-5231.1998 9784526 PMC108652

[B81] Shaffer S. R. Erondu A. I. Traboulsi C. Rai V. Krugliak Cleveland N. Israel A. (2021). Achieving Histologic Normalization in Ulcerative Colitis Is Associated with a Reduced Risk of Subsequent Dysplasia. Inflamm. Bowel Dis. izab130. 10.1093/ibd/izab130 PMC9122749 34037230

[B82] Shakya M. Lo C.-C. Chain P. S. G. (2019). Advances and Challenges in Metatranscriptomic Analysis. Front. Genet. 10, 904. 10.3389/fgene.2019.00904 31608125 PMC6774269

[B83] Shapiro H. Thaiss C. A. Levy M. Elinav E. (2014). The Cross Talk between Microbiota and the Immune System: Metabolites Take center Stage. Curr. Opin. Immunol. 30, 54–62. 10.1016/j.coi.2014.07.003 25064714

[B84] Sharma V. Rodionov D. A. Leyn S. A. Tran D. Iablokov S. N. Ding H. (2019). B-vitamin Sharing Promotes Stability of Gut Microbial Communities. Front. Microbiol. 10. 10.3389/fmicb.2019.01485 PMC6615432 31333610

[B85] Silva Y. P. Bernardi A. Frozza R. L. (2020). The Role of Short-Chain Fatty Acids from Gut Microbiota in Gut-Brain Communication. Front. Endocrinol. 11. 10.3389/fendo.2020.00025 PMC7005631 32082260

[B86] Singh N. Gurav A. Sivaprakasam S. Brady E. Padia R. Shi H. (2014). Activation of Gpr109a, Receptor for Niacin and the Commensal Metabolite Butyrate, Suppresses Colonic Inflammation and Carcinogenesis. Immunity 40, 128–139. 10.1016/j.immuni.2013.12.007 24412617 PMC4305274

[B87] Sobhani I. Tap J. Roudot-Thoraval F. Roperch J. P. Letulle S. Langella P. (2011). Microbial Dysbiosis in Colorectal Cancer (CRC) Patients. PLoS ONE 6, e16393. 10.1371/journal.pone.0016393 21297998 PMC3029306

[B88] Su G. Sun G. Liu H. Shu L. Zhang J. Guo L. (2015). Niacin Suppresses Progression of Atherosclerosis by Inhibiting Vascular Inflammation and Apoptosis of Vascular Smooth Muscle Cells. Med. Sci. Monit. 21, 4081–4089. 10.12659/MSM.895547 26712802 PMC4699630

[B89] Szamosi J. C. Forbes J. D. Copeland J. K. Knox N. C. Shekarriz S. Rossi L. (2020). Assessment of Inter-laboratory Variation in the Characterization and Analysis of the Mucosal Microbiota in Crohn's Disease and Ulcerative Colitis. Front. Microbiol. 11, 2028. 10.3389/fmicb.2020.02028 32973734 PMC7472644

[B90] The Integrative HMP iHMP Research Network Consortium (2014). The Integrative Human Microbiome Project: Dynamic Analysis of Microbiome-Host Omics Profiles during Periods of Human Health and Disease. Cell Host Microbe 16, 276–289. 10.1016/j.chom.2014.08.014 25211071 PMC5109542

[B91] Thomas A. M. Manghi P. Asnicar F. Pasolli E. Armanini F. Zolfo M. (2019). Metagenomic Analysis of Colorectal Cancer Datasets Identifies Cross-Cohort Microbial Diagnostic Signatures and a Link with Choline Degradation. Nat. Med. 25, 667–678. 10.1038/s41591-019-0405-7 30936548 PMC9533319

[B92] Ungaro R. Bernstein C. N. Gearry R. Hviid A. Kolho K.-L. Kronman M. P. (2014). Antibiotics Associated with Increased Risk of New-Onset Crohn's Disease but Not Ulcerative Colitis: a Meta-Analysis. Am. J. Gastroenterol. 109, 1728–1738. 10.1038/ajg.2014.246 25223575

[B93] Vich Vila A. Imhann F. Collij V. Jankipersadsing S. A. Gurry T. Mujagic Z. (2018). Gut Microbiota Composition and Functional Changes in Inflammatory Bowel Disease and Irritable Bowel Syndrome. Sci. Transl. Med. 10, eaap8914. 10.1126/scitranslmed.aap8914 30567928

[B94] Wang D. Mann J. R. Dubois R. N. (2005). The Role of Prostaglandins and Other Eicosanoids in the Gastrointestinal Tract. Gastroenterology 128, 1445–1461. 10.1053/j.gastro.2004.09.080 15887126

[B95] Weinstock J. V. Elliott D. E. (2009). Helminths and the IBD Hygiene Hypothesis. Inflamm. Bowel Dis. 15, 128–133. 10.1002/ibd.20633 18680198

[B96] Wirbel J. Pyl P. T. Kartal E. Zych K. Kashani A. Milanese A. (2019). Meta-analysis of Fecal Metagenomes Reveals Global Microbial Signatures that Are Specific for Colorectal Cancer. Nat. Med. 25, 679–689. 10.1038/s41591-019-0406-6 30936547 PMC7984229

[B97] Wishart D. S. (2016). Emerging Applications of Metabolomics in Drug Discovery and Precision Medicine. Nat. Rev. Drug Discov. 15, 473–484. 10.1038/nrd.2016.32 26965202

[B98] Yachida S. Mizutani S. Shiroma H. Shiba S. Nakajima T. Sakamoto T. (2019). Metagenomic and Metabolomic Analyses Reveal Distinct Stage-specific Phenotypes of the Gut Microbiota in Colorectal Cancer. Nat. Med. 25, 968–976. 10.1038/s41591-019-0458-7 31171880

[B99] Yang Y. Jobin C. (2017). Novel Insights into Microbiome in Colitis and Colorectal Cancer. Curr. Opin. Gastroenterol. 33, 422–427. 10.1097/MOG.0000000000000399 28877044 PMC5826583

[B100] Yvellez O. V. Rai V. Sossenheimer P. H. Hart J. Turner J. R. Weber C. (2021). Cumulative Histologic Inflammation Predicts Colorectal Neoplasia in Ulcerative Colitis: A Validation Study. Inflamm. Bowel Dis. 27, 203–206. 10.1093/ibd/izaa047 32152624 PMC7813748

[B101] Zelante T. Iannitti R. G. Cunha C. De Luca A. Giovannini G. Pieraccini G. (2013). Tryptophan Catabolites from Microbiota Engage Aryl Hydrocarbon Receptor and Balance Mucosal Reactivity via Interleukin-22. Immunity 39, 372–385. 10.1016/j.immuni.2013.08.003 23973224

[B102] Zhang X. Li L. Butcher J. Stintzi A. Figeys D. (2019). Advancing Functional and Translational Microbiome Research Using Meta-Omics Approaches. Microbiome 7, 154. 10.1186/s40168-019-0767-6 31810497 PMC6898977

[B103] Zhang Y. Thompson K. N. Branck T. Yan Yan Y. Nguyen L. H. Franzosa E. A. (2021a). Metatranscriptomics for the Human Microbiome and Microbial Community Functional Profiling. Annu. Rev. Biomed. Data Sci. 4, 279–311. 10.1146/annurev-biodatasci-031121-103035 34465175

[B104] Zhang Y. Thompson K. N. Huttenhower C. Franzosa E. A. (2021b). Statistical Approaches for Differential Expression Analysis in Metatranscriptomics. Bioinformatics 37, i34–i41. 10.1093/bioinformatics/btab327 34252963 PMC8275336

